# Does gold behaves as hydrogen? A joint theoretical and experimental study†

**DOI:** 10.1039/c9na00780f

**Published:** 2020-01-07

**Authors:** Zhengbo Qin, Jiangle Zhang, Chen Wang, Lin Wang, Zichao Tang

**Affiliations:** Anhui Province Key Laboratory of Optoelectric Materials Science and Technology, Department of Physics, Anhui Normal University Wuhu Anhui 241000 China wave0403@163.com; State Key Laboratory of Physical Chemistry of Solid Surfaces, Department of Chemistry, College of Chemistry and Chemical Engineering, Xiamen University Xiamen 361005 China zctang@xmu.edu.cn

## Abstract

It has been established that the noble-metal–H analogue has been found in a large number of noble-metal–ligand clusters in view of geometric and electronic structures. Here, we demonstrated a different view of noble-metal–H analogue between noble-metal and hydrogen in M(SCH_3_)_2_^−^ (M = Cu, Ag, Au and H) systems. Although H(SCH_3_)_2_^−^ is a typical ion-hydrogen bonding cluster dramatically different from the chemical bonding clusters of M(SCH_3_)_2_^−^ (M = Cu, Ag and Au), the comparison of the two typical bonding patterns has not yet been fully investigated. Through a series of chemical bonding analyses, it is indicated that the evolution has been exhibited from typical ionic bonding in Cu(SCH_3_)_2_^−^ to a significant covalent bonding nature in Au(SCH_3_)_2_^−^ and hydrogen bonding dominating in H(SCH_3_)_2_^−^. The comparison of M(SCH_3_)_2_^−^ (M = Cu, Ag and Au) with H(SCH_3_)_2_^−^ illustrates the differences in bonding between noble metals and hydrogen, which are mainly related to their diverse atomic orbitals participating in chemical bonding.

## Abstract 2

It has been established that noble-metal–H analogue has been found in plenty of noble-metal–ligand clusters in view of geometric and electronic structures. Now, it is beyond the analogy in the complexes of M(SCH_3_)_2_^−^ (M = Cu, Ag, Au and H).

## Graphical abstract

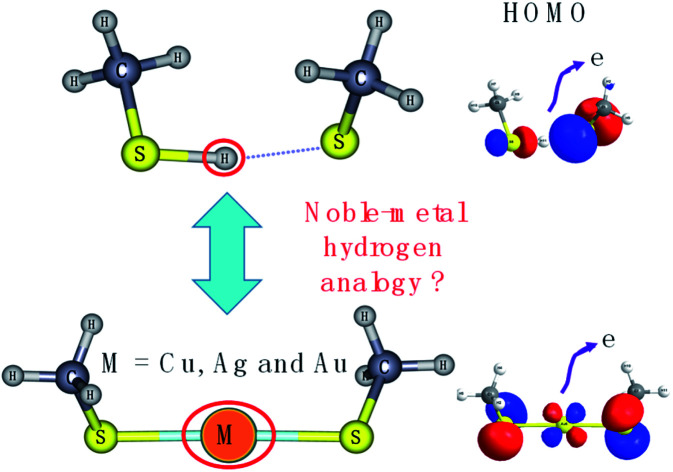

## 1. Introduction

Negative ion photoelectron velocity-map imaging (NI-PEVMI) spectroscopy is a potent and sensitive tool to give insight into the electronic properties of molecules with high energy resolution and high efficiency.^1^ NI-PEVMI allows the accurate measurement of electron affinity and vibrational frequencies, and provides direct understanding of the molecular orbital properties of anions in view of photoelectron angular distribution (PAD) analyses. In addition to detecting stable molecules, transient reaction intermediates, radicals and even transition states can be captured using the NI-PEVMI technique.^2–6^

Since noble-metal–hydrogen (especially for gold–hydrogen) analogy and isolobal analogy were discovered in extensive noble-metal–ligand complexes, widespread interests to understand the geometric structure and molecular orbital (MO) properties for the synthesis of various coordination-protected nanoclusters have increased. In 2003, H-doped gold clusters presented a spectral feature analogous to that of pure gold clusters.^7^ Recently, a similar phenomenon has also been investigated in Cu_*n*_H and Ag_*n*_H anion clusters.^8^ It is not a unique instance; similar structural and electronic properties and chemical bonding have been extended in many other clusters such as Au–Si, Au–B, Au–BX (X = F, Cl, Br) and Au–C.^9–18^ The idea with fundamental interest currently has potential application in designing and synthesizing coordination protected metal nanoclusters for special functions. Thus, these clusters with special electronic and geometric structures are explored and tailored for physical, chemical and optical stability to be incorporated into various applications.

In the recent two decades, the study on the interactions of thiols with gold surfaces and gold nanoparticles was one of the hottest research fields because thiolate decorated self-assembled monolayers (SAMs) on gold surfaces and gold nanoparticles (AuNPs) possess special stability under ambient environment.^19^ The thiolate stabilized gold SAMs and nanoparticles play vital roles in novel catalytic behavior and potential applications in nano-electronics and nano-sensors.^20^ Two milestone discoveries for thiolate protected SAMs and AuNPs are the gold-adatom mediating Au–S bond formation on gold surface and AuNP Au_102_(*p*-MBA)_44_ structural determination.^21,22^ Both of these reveal that two basic units, linear (RS–Au–SR) and (RS–Au–SR–Au–SR), on the surfaces have emerged as important bonding motifs to account for the stability. Additionally, these two binding motifs were isolated in the gas phase and subjected to the photoelectron spectroscopic characterization by Wang group.^23^ It has been found that ionic and covalent contributions from these motifs were almost equal. The disciplines unraveled in previous work prompt extensive thiolate protected noble-metal (group 11 metals) SAM and nanoparticle research, especially gold, and even for some thiolate protected silver and copper SAMs and nanoparticles.^24–35^

Although still progressing, the exception of gold–hydrogen analogy has also been found in the Au_7_H nanocluster in view of its catalytic capability with enhanced reactivity in contrast to the Au_8_ cluster.^36^ Fortified by the idea of violation of gold–hydrogen analogy, we extended the concept to their respective clusters (M(SCH_3_)_2_^−^ (M = Cu, Ag and Au) and H(SCH_3_)_2_^−^) and will discuss their geometrical, electronic and chemical bonding properties in the following section. The structural and electronic properties of these basic units reported in this work will shed light on a deeper understanding of the bonding interactions for the synthesis of these clusters.

## 2. Experimental details

The experimental apparatus has been reported in detail elsewhere.^37^ A brief introduction is as follows: the experiments were performed using our self-made NI-PEVMI spectroscope with a laser ablation source. The M(SCH_3_)_2_^−^ (M = Cu, Ag, Au and H) anions were produced *via* collision reactions of noble-metal atom with trace methanethiol in the presence of a supersonic beam of helium (99.999%) carrier gas. The anions were mass-selected using a time-of-flight mass spectrometer, guided to the photodetachment region, and subjected to photodetachment experiments by crossing with a laser beam (Nd:YAG laser). The resulting photoelectrons were imaged *via* a velocity map imaging photoelectron spectrometer and recorded by a charge-coupled device camera. Every NI-PEVMI spectrum was accumulated with 5 × 10^4^ to 10^5^ laser shots operated at 10 Hz. The original 3D distribution was reconstructed through the Basis Set Expansion (BASEX) inverse Abel transform method.^38^ Under current conditions, strong S^−^ provided a convenient NI-PEVMI calibrant along with noble-metal atom anions (Cu^−^, Ag^−^ and Au^−^). Typical energy resolution was about 3.0%, *i.e.*, 30 meV for 1 eV photoelectrons.

## 3. Computational details

All theoretical calculations were carried out using the Gaussian 09 package.^39^ The basis set of aug-cc-pVTZ-pp^40–42^ with relativistic effective small core pseudopotentials was used for copper, silver and gold atoms, and aug-cc-pVTZ^43^ for other atoms. The structures for the anionic and neutral complexes were optimized at the B3LYP, CAM-B3LYP and wB97XD theory.^44–48^ Vibration analysis was employed to check whether the optimized structures were at the true local minima or not. The excitation energies were obtained by TD-DFT calculations.^49^ The adiabatic detachment energy (ADE) was defined as the energy of the origin transition between the ground state of the anion and the neutral, which also represents the electron affinity of neutral species. The vertical detachment energy (VDE) was defined as the energy difference between the ground state of the anion and the ground state of the neutral at the anion geometry. Natural resonance theory (NRT)^50–52^ based on natural bond orbitals (NBO)^53,54^ method and electron localization function (ELF)^55^ analyses were performed to further understand the nature of the chemical bonding in the four species.

## 4. Results and discussion

### 4.1 NI-PEVMI spectra

**Fig. 1:**
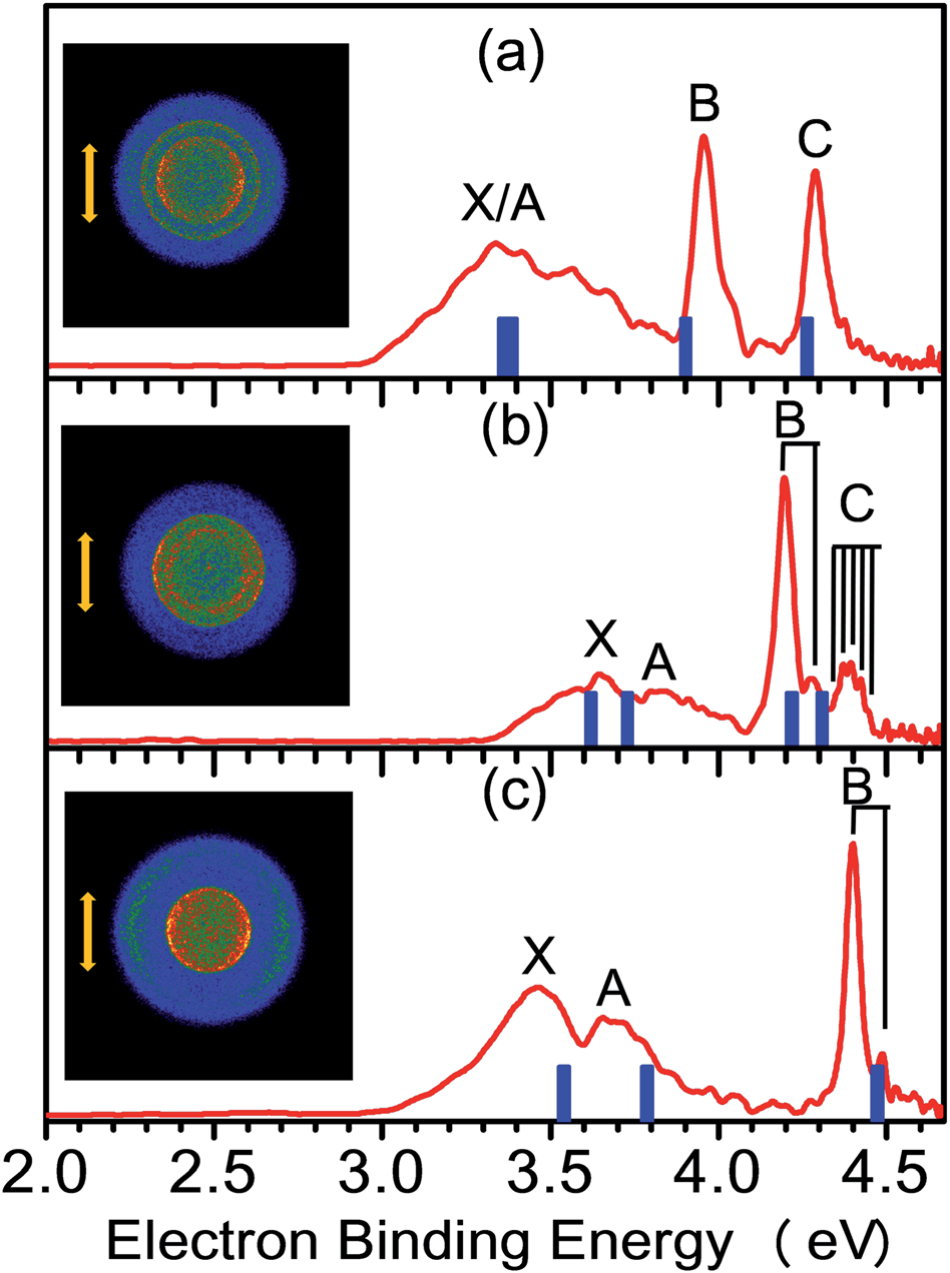
NI-PEVMI spectra of (a) Cu(SCH_3_)_2_^−^, (b) Ag(SCH_3_)_2_^−^, and (c) Au(SCH_3_)_2_^−^ at 266 nm (4.661 eV). Inset images represent the raw photoelectron image for each anion. The photoelectron bands in the experimental spectra are labeled as X, A, B, *etc.* The simulated stick spectra (blue vertical bars) are superimposed onto the experimental spectra (red). The double arrow indicates the directions of the laser polarization.

**Fig. 2:**
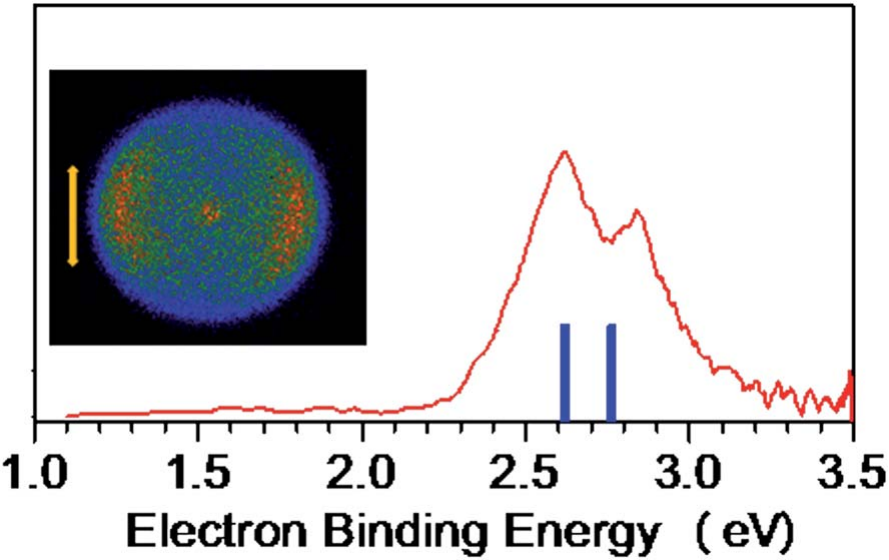
NI-PEVMI spectrum of CH_3_S^−^(CH_3_SH) at 355 nm (3.496 eV). Left column represents the raw photoelectron image. The simulated stick spectrum (blue vertical bars) is superimposed onto the experimental spectrum (red). The double arrow indicates the direction of the laser polarization.

The NI-PEVMI spectra of M(SCH_3_)_2_^−^ (M = Cu, Ag and Au) are displayed in Fig. 1. All of them exhibit a very broad feature for the first two bands X and A. In contrast, peaks B and C in Cu(SCH_3_)_2_^−^ and Ag(SCH_3_)_2_^−^ and peak B in Au(SCH_3_)_2_^−^ present much sharper property, as shown in the threshold photodetachment region. The adiabatic detachment energies (ADEs) were derived from the onset of feature X as 2.97 ± 0.03 eV for Cu(SCH_3_)_2_^−^, 3.32 ± 0.03 eV for Ag(SCH_3_)_2_^−^, and 3.06 ± 0.03 eV for Au(SCH_3_)_2_^−^. The first vertical detachment energies (VDE1) were determined as 3.34 ± 0.03 eV for Cu(SCH_3_)_2_^−^, 3.64 ± 0.03 eV for Ag(SCH_3_)_2_^−^, and 3.46 ± 0.03 eV for Au(SCH_3_)_2_^−^ from the maximum of band X. Moreover, vibrational progressions were observed in band B (653 ± 50 cm^−1^) and C (194 ± 50 cm^−1^) for Ag(SCH_3_)_2_^−^ and in band B (710 ± 50 cm^−1^) for Au(SCH_3_)_2_^−^. The NI-PEVMI spectrum of H(SCH_3_)_2_^−^ has been acquired at 355 nm as shown in Fig. 2. The ADE was measured from the onset of the first band X as 2.30 ± 0.03 eV and VDE was determined as 2.62 ± 0.03 eV. The ADEs and VDEs of all the observed bands are summarized in Table 1 and compared with theoretical calculations.

**Table:** Observed and calculated vertical (VDE) and adiabatic (ADE) detachment energies for M(SCH_3_)_2_^−^ (M = Cu, Ag, Au and H) at the level of CAM-B3LYP/aug-cc-pVTZ(-pp) including ZPEs. Experimentally measured anisotropic parameters *β* for M(SCH_3_)_2_^−^ (M = Cu, Ag, Au and H)

M	ADE [eV]	Feature	VDE [eV]		*β*
			Exp.	Theo.	
				TD-DFT	
Cu	2.97(3)	X	3.34(3)	3.36	−0.22
	2.90^a^	A	3.34(3)	3.39	
		B	3.96(3)	3.90	0.06
		C	4.29(2)	4.26	−0.32
Ag	3.32(3)	X	3.64(3)	3.62	−0.20
	3.26^a^	A	3.82(3)	3.73	
		B	4.20(2)	4.22	−0.19
		C	4.39(2)	4.31	0.05
Au	3.06(3)	X	3.46(3)	3.54	−0.26
	3.02^a^	A	3.70(3)	3.79	
		B	4.40(2)	4.47	−0.16
H	2.30(3)	X	2.62(3)	2.62	−0.42
	2.33^a^	A	2.84(2)	2.76	

a Theoretical result.

### 4.2 Structural calculations and spectral assignments

**Fig. 3:**
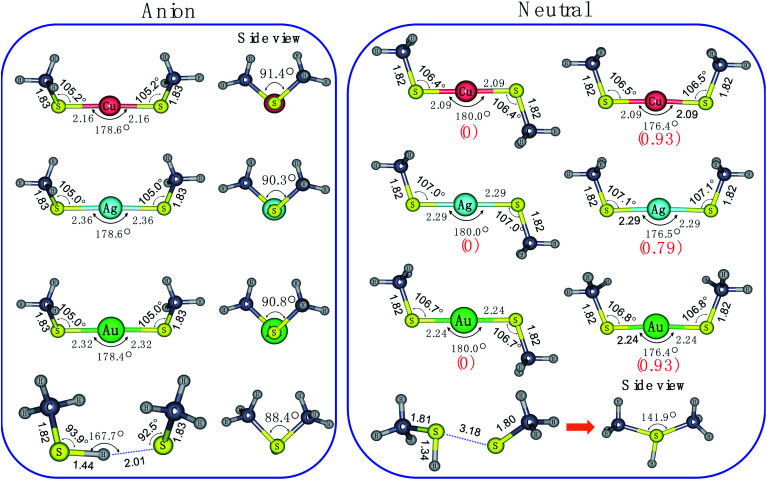
Optimized structures for anionic and neutral species of the M(SCH_3_)_2_^−^ (M = Cu, Ag, Au and H) at the CAM-B3LYP/aug-cc-pVTZ(-pp) level. Relative energies (in kJ mol^−1^), which were evaluated at the same level of theory including ZPEs are given in parentheses.

To aid in understanding the observed PE-VMI spectra for these species, we performed density functional theory (DFT) calculations on the various possible structures. As displayed in Fig. 3, the most stable anion geometries, M(SCH_3_)_2_^−^ (M = Cu, Ag and Au), have *C*_2_ symmetry with quasi-linear S–M–S bond and the dihedral angle of C–S–S–C is near 90°. However, for H(SCH_3_)_2_^−^, S–H⋯S H-bond is formed and the *θ*(S–H⋯S) = 167.7° at the CAM-B3LYP level. It was also indicated that there were two distinct bonding motifs in M(SCH_3_)_2_^−^ (M = Cu, Ag and Au) and H(SCH_3_)_2_^−^. Compared with the directional nature of covalent or ionic bonding (linear S–M–S) in M(SCH_3_)_2_^−^ (M = Cu, Ag and Au), the H-bond exhibits a bending feature in H(SCH_3_)_2_^−^. Upon photodetachment, for neutral M(SCH_3_)_2_ (M = Cu, Ag and Au), the dihedral angle of C–S–S–C varied from ∼90° to 180° in the form of *trans*-conformer with *C*_2h_ symmetry and to 0° in the form of *cis*-conformer with *C*_2v_ symmetry.

To better understand the broad feature of band X, we performed Franck–Condon simulations based on harmonic oscillator approximation on these three cases. As revealed in Fig. S1,† for all of them, CS–M–SC torsion mode, S–M–S stretching mode and S–M–S bending mode make primary contributions to the broad spectral band X. This situations are consistent with relatively large geometric variations in dihedral angle of C–S–S–C and S–M–S bond exhibited in Fig. 3.

**Fig. 4:**
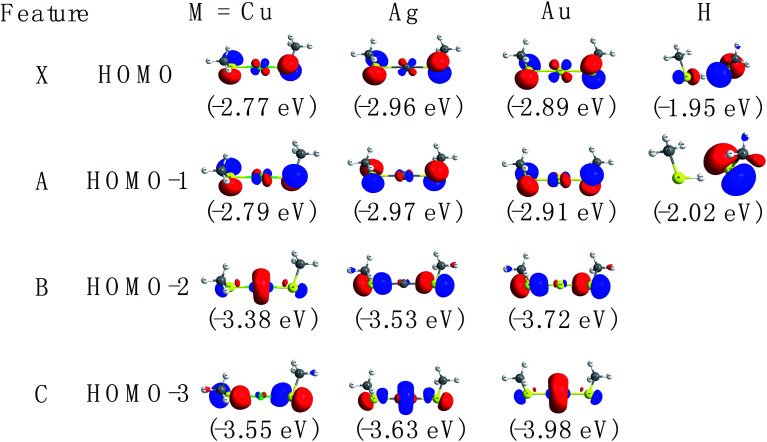
The contour plots of the occupied valence orbitals of the ground state of M(SCH_3_)_2_^−^ (M = Cu, Ag, Au and H) calculated at the CAM-B3LYP/aug-cc-pVTZ(-pp) level (isocontour = 0.06 a.u.) (blue and red colors represent different phases of molecular orbital).

The spectral patterns in the experiment can be glimpsed from the detached molecular orbitals (MOs) for these species. The calculated Kohn–Sham molecular orbitals are shown in Fig. 4. Combining frontier MO nature with photoelectron angular distributions (PADs), it is revealed that for bands X and A, negative anisotropy parameters for four species in Fig. 1 (also Table 1) are mainly associated with the p-type orbital photodetachment and with minor d-type orbital photodetachment for metal counterparts, as shown in Fig. 4 (*β* = −0.22 for Cu, *β* = −0.20 for Ag and *β* = −0.26 for Au). It is interesting to note that the isotropic PAD distributions (*β* ∼ 0) observed for peak B in Cu(SCH_3_)_2_^−^ and peak C in Ag(SCH_3_)_2_^−^ are associated with significant d-type (high angular momentum quantum number) orbital contributions (48% Cu 3d in HOMO−2 and 26% Ag 4d in HOMO−3), as listed in Table S3.† The higher binding energy peaks (B and C for Cu(SCH_3_)_2_^−^ and Ag(SCH_3_)_2_^−^, and B for Au(SCH_3_)_2_^−^) come from electron detachment, primarily because of the nonbonding noble metal *n*d orbitals and S 3p_*z*_ orbital. Thus, they display relatively sharp peaks with good Franck–Condon overlaps between anion and neutral forms upon photodetachment. Thus, NI-PEVMI with the aid of PAD analysis is a good sign for the detailed spectral assignment in this work. The natural atomic orbitals for M(SCH_3_)_2_^−^ (M = Cu, Ag, Au and H) are listed in Table S3 (see ESI†).

### 4.3 Discussion

Chemical bonding analyses based on NBO theory have been carried out to understand the two typical bonding motifs in M(SCH_3_)_2_^−^ (M = Cu, Ag, Au and H) systems. As can be seen from Table 2, the positive charge distribution on noble-metal atoms bears out the evolution from large positive charge (+0.287*e*) on Cu to the nearly zero charge (+0.076) on Au. The difference in charge distribution on various noble-metal atoms indicates possible chemical bonding evolution from Cu to Au species, which can be confirmed in following results. For H in S–H⋯S H-bond, the charge distribution is +0.162*e*. H-bonding of S–H⋯S involves modest electron transfer (∼0.22*e*) from CH_3_S^−^ to CH_3_SH moiety, essentially corresponding to the occupied S 3p electron in CH_3_S^−^ transferring to the S–H σ* orbital, as shown in Fig. 4 and Table S3.† The interaction of H-bond is clearly elucidated from the NBO donor–acceptor orbital pair characterization in Fig. 6. The principle donor orbital is the sulfur p-type orbital in the CH_3_S^−^ moiety that is oriented appropriately to interact with the S–H bond in the CH_3_SH moiety. The corresponding principle acceptor orbital possesses anti-bonding σ* character on the S–H bond. The NBO analysis calculated at the CAM-B3LYP/aug-cc-pVTZ level suggests that the second order interaction energy *E*^(2)^ is 65.8 kcal mol^−1^. The charge transfer (CT) interaction leads to a 0.1 Å lengthening of the S–H distance compared to that in free CH_3_SH molecule (Fig. S2†).

Theoretical atomic net charges, M–S bond orders and natural localized molecular orbitals (NLMO) in M(SCH_3_)_2_^−^ (M = Cu, Ag, Au and H) at the CAM-B3LYP/aug-cc-pVTZ(-pp) level

**Table:** 

Charge population			
Species	NPA (M)	NPA (S)	NPA (–CH_3_)^a^
Cu(SCH_3_)_2_^−^	0.287	−0.466	−0.155
Ag(SCH_3_)_2_^−^	0.269	−0.485	−0.160
Au(SCH_3_)_2_^−^	0.076	−0.384	−0.154
H(SCH_3_)_2_^−^	0.162	−0.250/(−0.587)^a^	−0.128/(−0.198)^a^

a The number in parentheses denotes the charges in the CH_3_S^−^ group.

**Table:** 

Bond order				
Species	Bond	Wiberg	Mayer	NAO
Cu(SCH_3_)_2_^−^	Cu–S	0.650	0.736	0.711
Ag(SCH_3_)_2_^−^	Ag–S	0.585	0.884	0.626
Au(SCH_3_)_2_^−^	Au–S	0.684	1.114	0.692
H(SCH_3_)_2_^−^	CH_3_S–H	0.740	0.693	0.608
	S–H⋯S	0.241	0.296	0.262

**Table:** 

Natural localized molecular orbitals (NLMO)	
Bond	Component
Cu–S	85.3% S(sp^3.08^) + 14.7% Cu(sp^1.08^d^0.08^)
Ag–S	86.2% S(sp^4.39^) + 13.8% Ag(sp^1.08^d^0.08^)
Au–S	81.8% S(sp^4.56^) + 18.2% Au(sd^0.2^)
CH_3_S–H	65.0% S(sp^4.26^) + 35.0% H(s)

**Fig. 5:**
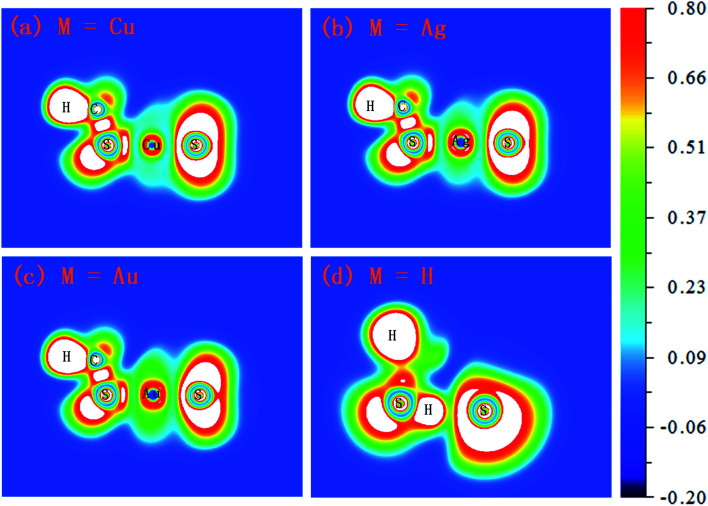
The electron localization function (ELF) isosurfaces (contour lines) for M(SCH_3_)_2_^−^ (M = Cu, Ag, Au and H) complexes (unit: a.u.).

**Fig. 6:**
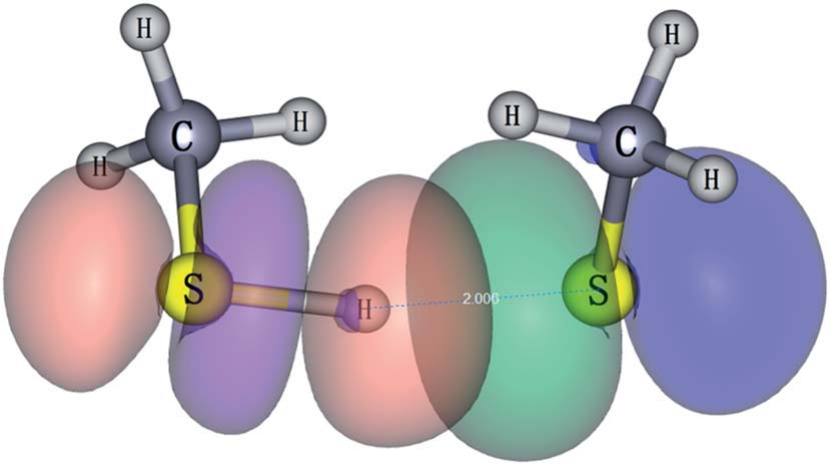
Natural bonding orbital interaction responsible for the hydrogen bond in CH_3_S^−^(CH_3_SH) (isovalue = 0.03).

The bond order increases from Cu and Ag to Au in the Wiberg and Mayer bond order analyses. Compared to the ionic and covalent bonds in M(SCH_3_)_2_^−^ (M = Cu, Ag and Au), the bond order of H-bond in H(SCH_3_)_2_^−^ (0.241 in Wiberg, 0.296 in Mayer and 0.262 in NAO as listed in Table 2) is considerably weaker than that in normal chemical bond (>0.5). NRT based on NBO theory bifurcates binding energy into covalent and ionic components. Therefore, M–S bonds are more ionic than covalent in Cu and Ag counterparts (the ionic component ≥70%) and Au–S bond in Au(SCH_3_)_2_^−^ has significant covalent character (≥43%), in accordance with previously reported results.^23^ Overall, in the NRT scheme, the covalent character decreases in the order of Au > Ag > Cu. For H(SCH_3_)_2_^−^, the covalent nature dominates in the S–H bond in the CH_3_SH moiety (69.90%), and the H-bond in the S–H⋯S moiety typically resembles ionic character (90.17%) (Table 3), but the strength is much weaker than that in a normal chemical bond.

**Table:** Summary of natural resonance theory (NRT) and covalent and ionic electrovalent contributions of the optimized structures of M(SCH_3_)_2_^−^ (M = Cu, Ag, Au and H) species at the CAM-B3LYP/aug-cc-pVTZ(-pp) level

Bond	T(NRT)^a^	NRT bond order	% Covalent	% Ionic
Cu–S	0.4959	0.1431c + 0.3528i	28.86	71.14
Ag–S	0.4956	0.1489c + 0.3466i	30.04	69.96
Au–S	0.4738	0.2057c + 0.2680i	43.41	56.59
CH_3_S–H	0.8907	0.6226c + 0.2681i	69.90	30.10
S–H⋯S	0.0956	0.0094c + 0.0862i	9.83	90.17

a Total NRT bond order is the sum of covalent (c) plus ionic (i) bond order.

For a given molecule, the region of strong electron localization corresponding to chemical bonds and free electron pairs can be visualized using the electron localization function (ELF) approach. As can be illustrated in Fig. 5, the Cu–S bond in Cu(SCH_3_)_2_^−^ and Ag–S bond in Ag(SCH_3_)_2_^−^ reflect significant ionic character due to the deep valley between the two atoms and bonding electrons being shifted towards the sulfur atom. Compared to the Cu–S bond in Cu(SCH_3_)_2_^−^, the Ag–S bond has slightly less ionic character. Notice that a significant electron pair density found for the Au–S bond in Au(SCH_3_)_2_^−^ showed the strongest covalent character amongst the three complexes. For H(SCH_3_)_2_^−^, the S–H bond in the CH_3_SH moiety possesses a typical covalent character. Nevertheless, the S–H⋯S H-bond demonstrates relatively small electron pair density in contrast to the S–H bond.

Although all of them have similar chemical formula, other than M(SCH_3_)_2_^−^ (M = Cu, Ag and Au) species, which can usually be written as (CH_3_S–M–SCH_3_)^−^, H(SCH_3_)_2_^−^ actually has the formula as CH_3_S^−^(CH_3_SH). Essentially, single hydrogen atom cannot participate in multiple chemical bonding as noble-metal atoms do to form sp^*x*^, sd^*y*^ or sp^*x*^d^*y*^ hybrid orbitals.

## 5. Conclusions

In this work, we report systematic research work on M(SCH_3_)_2_^−^ (M = Cu, Ag and Au) and H(SCH_3_)_2_^−^ complexes. The spectra of these complexes were obtained by NI-PEVMI. It reveals that density functional theory (DFT) calculations confirm the different bonding feature between Au, Ag, Cu and H counterparts. The results unraveled in this work could be useful for the design of other compounds and could provide new opportunities for the applications of M–SR clusters in catalysis or in the activation of small molecules.

In conclusion, NI-PEVMI provides the first systematically spectroscopic, geometric and chemical bonding characterization for the M(SCH_3_)_2_^−^ ions. Analyses of these NI-PEVMI spectra unravel details of the MO natures between noble-metal ligand complexes M(SCH_3_)_2_^−^ and the CH_3_S^−^(HSCH_3_) cluster in this most fundamental building block units. A joint study *via* NI-PEVMI and quantum chemical method is suitable for the EA, VDE and electronic structure predictions of these basic clusters. The results revealed in this work will shed light on the further exploration of thiolate protected metal nanoclusters in other systems.

## Conflicts of interest

The authors declare no competing financial interest.

## Supplementary Material

NA-002-C9NA00780F-s001
