# Supplementary material for: Does gold behaves as hydrogen? A joint theoretical and experimental study
Source: Nanoscale Adv. 2020 Jan 7;2(2):844–50. doi: 10.1039/c9na00780f (PMC9418258; doi:10.1039/c9na00780f)
Supplement: NA-002-C9NA00780F-s001 [file NA-002-C9NA00780F-s001.pdf]

Electronic Supplementary Material (ESI) for Nanoscale Advances.
This journal is © The Royal Society of Chemistry 2020

Supplementary Information for

Gold behaves as hydrogen? A joint theoretical and experimental study

Zhengbo Qin[1, *], Jiangle Zhang[1, 2], Chen Wang[1], Lin Wang[1], and Zichao Tang[2, *]

[1]Anhui Province Key Laboratory of Optoelectric Materials Science and Technology, Department of Physics, Anhui Normal University, Wuhu, Anhui 241000, China

[2]State Key Laboratory of Physical Chemistry of Solid Surfaces, Department of Chemistry, College of Chemistry and Chemical Engineering, Xiamen University, Xiamen 361005, China

Corresponding author: wave0403@163.com; zctang@xmu.edu.cn;

Table S1: Observed and calculated vertical (VDE) and adiabatic (ADE) detachment energies for $M(SCH_3)_2^-$ (M = Cu, Ag, Au and H) at the level of B3LYP/aug-cc-pVTZ(-pp) including ZPEs.

**M**	**ADE [eV]**	**Feature**	**VDE [eV]**	
			Exp.	Theo. TD-DFT
**Cu**	2.97(3)	X	3.34(3)	3.07
	2.69[a]	A	3.34(3)	3.10
		B	3.96(3)	3.61
		C	4.29(2)	3.97
**Ag**	3.32(3)	X	3.64(3)	3.28
	2.98[a]	A	3.82(3)	3.39
		B	4.20(2)	3.88
		C	4.39(2)	3.97
**Au**	3.06(3)	X	3.46(3)	3.26
	2.81[a]	A	3.70(3)	3.51
		B	4.40(2)	4.19
**H**	2.30(3)	X	2.62(3)	2.72
	2.39[a]	A	2.84(2)	2.86

[a] Theoretical result.

Table S2: Observed and calculated vertical (VDE) and adiabatic (ADE) detachment energies for $M(SCH_3)_2^-$ (M = Cu, Ag, Au and H) at the level of wB97XD/aug-cc-pVTZ(-pp) including ZPEs.

**M**	**ADE [eV]**	**Feature**	**VDE [eV]**	
			Exp.	Theo. TD-DFT
**Cu**	2.97(3)	X	3.34(3)	3.28
	2.89[a]	A	3.34(3)	3.31
		B	3.96(3)	3.82
		C	4.29(2)	4.18
**Ag**	3.32(3)	X	3.64(3)	3.62
	3.26[a]	A	3.82(3)	3.73
		B	4.20(2)	4.22
		C	4.39(2)	4.31
**Au**	3.06(3)	X	3.46(3)	3.54
	3.00[a]	A	3.70(3)	3.79
		B	4.40(2)	4.47
**H**	2.30(3)	X	2.62(3)	2.67
	2.36[a]	A	2.84(2)	2.81

[a] Theoretical result.

Table S3: Natural atomic orbital analyses for the ground state of $M(SCH_3)_2^-$ (M = Cu, Ag, Au and H) at the CAM-B3LYP/aug-cc-pVTZ(-pp) level.

Species	MO	Contribution from M, S and H atomic orbitals
M = Cu	HOMO	38% S(1) 3p + 17% Cu 3d + 38% S(2) 3p
	HOMO-1	38% S(1) 3p + 17% Cu 3d + 38% S(2) 3p
	HOMO-2	11% S(1) 3p + 48% Cu 3d + 25% Cu 4s + 11% S(2) 3p
	HOMO-3	42% S(1) 3p + 6% Cu 3p + 42% S(2) 3p
M = Ag	HOMO	42% S(1) 3p + 10% Ag 4d + 41% S(2) 3p
	HOMO-1	42% S(1) 3p + 10% Ag 4d + 42% S(2) 3p
	HOMO-2	32% S(1) 3p + 11% Ag 4p + 32% S(2) 3p
	HOMO-3	19% S(1) 3p + 26% Ag 4d + 27% Ag 5s + 19% S(2) 3p
M = Au	HOMO	38% S(1) 3p + 14% Au 5d + 1% Au 6s + 38% S(2) 3p
	HOMO-1	38% S(1) 3p + 14% Au 5d + 1% Au 6s + 38% S(2) 3p
	HOMO-2	6% S(1) 3s + 28% S(1) 3p + 17% Au 5p + 6% S(2) 3s + 28% S(2) 3p
	HOMO-3	9% S(1) 3p + 24% Au 5d + 51% Au 6s + 9% S(2) 3p
M = H	HOMO	77% S(1) 3p[a] + 1% H 1s + 2% C 2p + 17% S(2) 3p
	HOMO-1	92% S(1) 3p

[a] The S denotes the sulfur atom in CH_3S^- group.

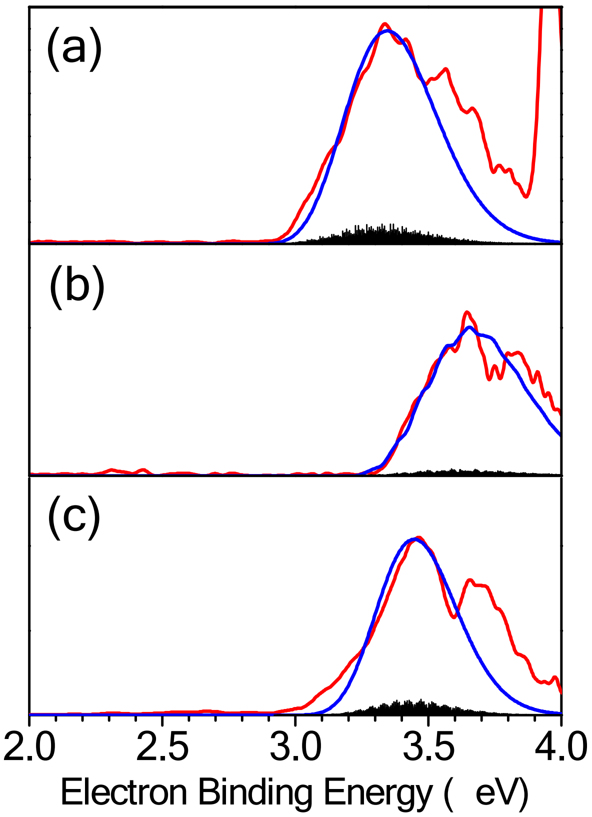

Figure S1. Franck-Condon simulations using the PESCAL program[S1] for the photodetachment in first spectral band X: (a) $Cu(SCH_3)_2^-$, (b) $Ag(SCH_3)_2^-$, and (c) $Au(SCH_3)_2^-$. Red curves stand for the experimental data, blue curves for Franck-Condon (FC) simulated results, and black sticks for simulated vibronic transitions. The individual vibrational peaks contours are approximated as a Gaussian function with 0.01 eV FWHM (full width at half-maximum). And the simulated vibrational temperature of ~100 K was adopted for anions.

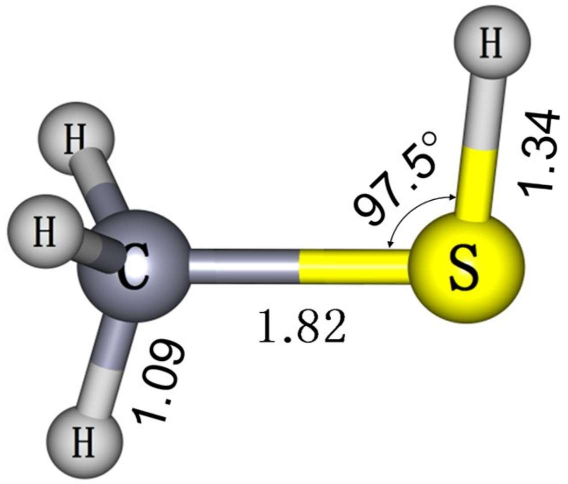

Figure S2. Optimized structures for CH_3SH at the CAM-B3LYP/aug-cc-pVTZ(-pp) level.

Table S4 Cartesian coordinates of $Cu(SCH_3)_2^-$, $Ag(SCH_3)_2^-$, $Au(SCH_3)_2^-$, $H(SCH_3)_2^-$ and CH_3SH calculated at the level of CAM-B3LYP/aug-cc-pVTZ(-PP).

**Symmetry C2**	**$Cu(SCH_3)_2^-$**		
C	0.000000000	2.938198000	-0.922469000
H	1.052800000	3.019844000	-0.658290000
S	-0.927525000	1.950137000	0.309631000
H	-0.071355000	2.505021000	-1.918598000
H	-0.422858000	3.942296000	-0.955653000
C	0.000000000	-2.938198000	-0.922469000
H	-1.052800000	-3.019844000	-0.658290000
S	0.927525000	-1.950137000	0.309631000
H	0.071355000	-2.505021000	-1.918598000
H	0.422858000	-3.942296000	-0.955653000
Cu	0.000000000	0.000000000	0.283673000

**Symmetry C2**	**$Ag(SCH_3)_2^-$**		
C	-3.051445000	-0.982936000	-0.607129000
H	-2.892345000	-0.729319000	-1.653698000
S	-2.305819000	0.260626000	0.509125000
H	-2.644949000	-1.977114000	-0.429546000
H	-4.125601000	-1.016664000	-0.424166000
C	3.051340000	-0.983206000	0.606871000
H	2.891489000	-0.730403000	1.653520000
S	2.305864000	0.260816000	-0.508975000
H	2.645375000	-1.977430000	0.428334000
H	4.125626000	-1.016352000	0.424548000
Ag	0.000007000	0.231938000	0.000003000

**Symmetry C2**	**$Au(SCH_3)_2^-$**		
C	0.000000000	3.075636000	-1.032956000
H	1.054643000	3.133738000	-0.771635000
S	-0.945010000	2.115497000	0.203529000

H	-0.087375000	2.641513000	-2.026841000
H	-0.405337000	4.087242000	-1.060917000
Au	0.000000000	0.000000000	0.172168000
C	0.000000000	-3.075636000	-1.032956000
H	-1.054643000	-3.133738000	-0.771635000
S	0.945010000	-2.115497000	0.203529000
H	0.087375000	-2.641513000	-2.026841000
H	0.405337000	-4.087242000	-1.060917000

**Symmetry C1**	**$H(SCH_3)_2^-$**		
C	-2.026059000	-0.772288000	0.712035000
H	-2.340365000	-1.712769000	0.255288000
S	-1.612856000	0.479638000	-0.551428000
H	-1.159415000	-0.980521000	1.343833000
H	-2.835373000	-0.430699000	1.359942000
C	1.948968000	-0.858966000	-0.662477000
H	2.192042000	-1.782251000	-0.139442000
S	1.646354000	0.498332000	0.510352000
H	1.035424000	-1.003369000	-1.237560000
H	2.764597000	-0.612180000	-1.339827000
H	0.269683000	0.661788000	0.117648000

**Symmetry Cs**	**CH_3SH**		
C	-0.047838000	1.151235000	0.000000000
H	0.430779000	1.547185000	0.890515000
S	-0.047838000	-0.663784000	0.000000000
H	0.430779000	1.547185000	-0.890515000
H	-1.090616000	1.457020000	0.000000000
H	1.281501000	-0.838246000	0.000000000

References

S1. K. M. Ervin, T. M. Ramond, G. E. Davico, R. L. Schwartz, S. M. Casey, W. C. Lineberger, J. Phys. Chem. A 2001, **105**, 10822–10831.
